# Serum vitamin D in hypertensive patients versus healthy controls is there an association?

**Published:** 2016

**Authors:** Roghayeh Akbari, Bahram Adelani, Reza Ghadimi

**Affiliations:** 1 Department of Internal Medicine, Babol University of Medical Sciences, Babol, Iran.; 2 Social Determinants of Health Research Center, Health Research Institute, Babol University of Medical Sciences, Babol, Iran.

**Keywords:** Blood pressure, Hypertension, Vitamin D, Calcium, PTH

## Abstract

**Background:**

Both vitamin D deficiency and hypertension are prevalent in the general population. Several observations indicate an association between vitamin D deficiency and high blood pressure. The present case-control study aimed to compare serum 25-hydroxyvitamin D (25-OHD) in hypertensive patients versus healthy controls.

**Methods:**

One hundred patients aged 30-60 years with hypertension (HTN) and 100 healthy controls without history of hypertention were compared regarding serum 25-OHD. Blood pressure was measured using standard method and the systolic and diastolic blood pressure more than140 mmHg and 90 mmHg respectively were considered as HTN. Patients and controls with coexistent morbidities, vitamin D supplementation were excluded. The serum levels of 25-OHD, PTH and calcium were measured after obtaining a written informed consent from the patients and taking their blood pressure under standard conditions. In statistical analysis, the two groups were compared using independent t test and chi-square test using SPSS Version 18.

**Results:**

The mean age of patients and controls was comparable (53.7±6.4 vs 52.3±7.54 years, P=0.17). Serum 25-OHD in HTN was significantly higher than controls (P=0.001).

**Conclusion:**

In the present study, serum 25-OHD level in hypertension was higher than controls. The results contradict with earlier studies indicating an association of HTN with vitamin D deficiency. This issue warrants further investigations in particular the follow-up of serum 25-OHD deficient and sufficient subjects with regard to the development of HTN.

Vitamin D deficiency is highly prevalent in general populations across various geographical regions and its contribution in the development of many common clinical conditions is of particular importance (1-4). According to earlier studies it has been estimated that 24-70% of the aging population have vitamin D (vit D) deficiency (2, 5). Vit D deficiency is more prevalent in Europe and America. In the Middle East and Islamic countries, habitual use of clothing covering most of the skin seems to have contribution in the development of vit D deficiency (6, 7). The effect of vitamin D is not limited to skeletal problem but because of its wide distribution across various body cells, vit D plays a different role in the health, survival and reproduction of humans (8). Several studies have emphasized its role in the prevention of diseases such as heart disease, cancer, inflammatory bowel disease, multiple sclerosis, rheumatoid arthritis, type 1 diabetes, pre-eclampsia, autoimmune and infectious diseases (3, 9-12).

Today, high blood pressure is considered as a worldwide problem and several studies have been conducted in respect to serum level of 25-hydroxy vitamin D3 and hypertension (3, 4, 13). Vit D has an inhibitory effect on the synthesis of renin (converting angiotensin I to II) or on the regulation of inflammation in laboratory animals (14, 15). Vitamin D also has the ability of adjusting the gene expression of natriuretic receptor (with the mechanism of hypotension and cardiac output via the kidney) (16).

However, the relationship between vit D and blood pressure across various studies is controversial (17). Also both vitamin D deficiency and hypertension are prevalent in the geographical region of this study. It is not clear whether the presence of these conditions in one subject is a cause and effect or incidental coexistent of these two common conditions. For these reasons, the present study was conducted to investigate the relationship between serum vitamin D level and hypertension (HTN) in hypertensive subjects presented to a university affiliated teaching hospital in Babol, North of Iran.

## Methods

This case-control study was conducted on 100 hypertensive patients aged 30-60 years at the teaching hospitals of Babol University of Medical Sciences and 100 age matched and sex-matched healthy subjects based on inclusion and exclusion criteria in 2013. Systolic and diastolic blood pressure greater than 140 mmHg and 90 mmHg, respectively were considered as the presence of HTN. All patients were selected consecutively according to inclusion criteria.

The subjects of the control group were selected among the patient’s relatives whit no history of hypertension. Subjects with pregnancy, known as renal disease, hypertension secondary to renal disease, inflammatory arthritis, taking diuretic medications and any supplements of vitamin D and calcium supplementation in the last year were excluded.

After taking the written informed consent from all subjects, blood pressure was measured under standard conditions after at least 10 minutes of rest in a quiet environment and for two occasions for at least a 15-minute interval. Serum vitamin D was assessed by measurement of 25-hydroxyvitamin D (25-OHD) level by ELISA methods. In addition, serum calcium and parathyroid hormone (PTH) were measured using the kit provided by German Merck Company according to manufacturer’s instruction. Serum vitamin D level was classified as deficient (less than 10 ng/ml), insufficient (10 to 30 ng/ml) and sufficient (more than 30 ng/ml).

All data were statistically analyzed by t-test and chi-square test using SPSS Version 15, and p<0.05 was considered as significant level.

## Results

All patients and controls completed the study. Gender distribution was similar in both the comparison group by 41% males and 59% females (mean age of patients and controls was comparable (53.7±6.4 and 52.3±7.4 years) respectively. There was no significant difference in the mean age of men and women between the two groups. Data according to sex in both patients and controls are presented in table 1. 

**Table 1:** Comparison of the mean of blood pressure, serum levels of vitamin D, PTH and calcium in patients with hypertension and healthy controls according to sex

		**Systolic ** **blood pressure (mmHg)**	**Diastolic blood pressure (mmHg)**	**Serum vitamin D** ** (ng/ml)**	**P-value**	**Serum PTH (pg/ml)**	**P-value**	**Serum Ca (mg/ml) **	**P-value**
Male	Patients	143.6±19.72	86.4±10.75	27.16±19.21	0.001	58.2±42.9	0.988	9.4±0.5	0.988
	Controls	115.6±9.36	75.97±7.68	17.97±9.17		59.6±29.1		9.4±0.5	
Female	Patients	147.6±19.09	112.45±12.64	23.64±19.54	0.001	76.8±37.3	0.002	9.3±0.6	0.254
	Controls	109±10.26	73.27±10.28	13.5±13.75		58.9±26		9.4±0.4	
Total	Patients	145.1±19.3	105.9±11.4	24.5±19.4	0.001	71.1±39.4	0.007	9.35±0.6	0.334
	Controls	112.3±10.3	74.3±9.3	15.3±12.2		59.2±27.1		9.43±0.52	

As shown in table 1, the mean of PTH serum level in two groups showed no significant difference between the men of the two groups; however, this difference was significant between the females of both groups. Serum vitamin D level was significantly higher in patients with HTN than healthy group (P=0.001). 

Proportion of serum 25-OHD deficiency, insufficiency and sufficiency in patients were 27%, 40% and 33% and in the control group 40%, 53% and 7% respectively. In table 2, the mean of systolic and diastolic blood pressure is presented according to serum vit D levels.

In table 3, the mean levels of vitamin D, calcium and PTH are given in both groups according to age group. According to the studies, the lower concentration of serum vitamin D and PTH in hypertensive patients was observed only in age ranging from 45 to 60 years.

**Table 2:** The mean of systolic and diastolic blood pressure of subjects based on the separation of serum level of vitamin D

**Mean of blood pressure(mmHg)**	**Group**	**Vitamin D Deficiency** **(≤** **10 ng/ml)**	**Vitamin D Insufficiency** **(10- 30ng/ml)**	**Normal Vitamin D** **(≥** **30 ng/ml)**
Systolic blood pressure	Patients	143.7±18	145.8±18.2	148.3±21.8
	Controls	110.7±12	116.6±10.3	111.4±12.1
Diastolic blood pressure	Patients	98.1±7.1	105.3±9.1	112.7±13.4
	Controls	72.4±11.2	76.2±7.4	71.4±8.9

**Table 3:** Comparison of the serum levels of vitamin D, calcium and PTH inpatients with hypertension and normal subjects according to age group

**Age group**		**Serum vitamin D** **(ng/ml)**	**P-value**	**Serum PTH** **(pg/ml)**	**P value**	**Serum Ca** **(mg/ml)**	**P-value**
30-45 years	Patients	19.9±17.2	0.9	83.4±41.6	0.3	8.9±0.9	0.31
	Controls	20.9±25.6		71.3±30.4		9.1±0.4	
45-60 years	Patients	28.5±29.7	0.001	83.9±54	0.001	10.4±9.7	0.9
	Controls	15.3±11.9		56.7±25.9		10.6±10.3	

## Discussion

The results of this study indicate a higher level of serum 25-OHD in HTN compared with the healthy controls. These findings are inconsistent with previous studies which have shown an association between vit D deficiency and HTN (18-21). Higher prevalence of HTN in African Americans may be partly attributed to lower serum vitamin synthesis due to skin pigmentation (18).

 Similarly, Martins et al. (19) in 2007 in a study on 15088 people found significantly lower mean 25-OHD in patients with HTN as compared with healthy controls. In another study, the prevalence of HTN in subjects with sufficient vit D was 20% lower as compared with those with vit D deficiency (20). 

A significant inverse correlation was observed between serum vit D level and high blood pressure of white people based on the data of National Health and Nutrition Examination Survey (NHANESIII) (20).

**Figure 1:**
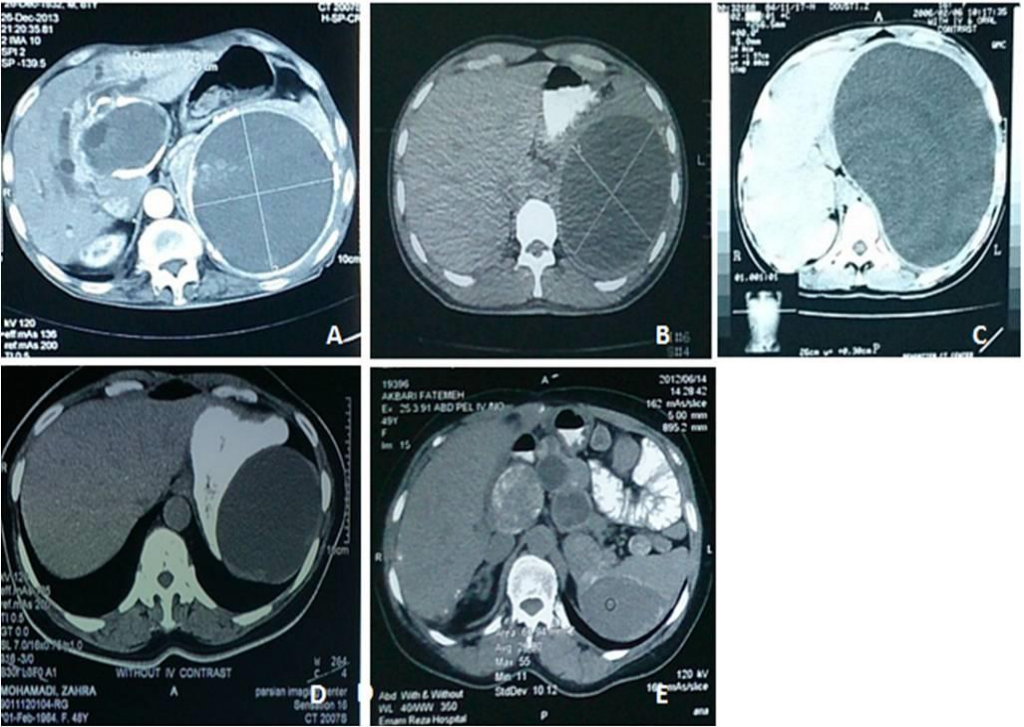
remarkable CT-scan findings in our trial;

A. 81 year-old male with gallstone (diameter of 11 mm) and multi-cystic lesions with diffuse calcification in liver (maximum diameter of 85 mm) and spleen (maximum diameter of 143 mm).

B. 33 year-old male with non-enhanced hypodense lesion (110*124 mm) suggestive for hydatid splenic cyst.

C. 16 year-old female with huge well-defined cystic lesion with diameter of 200mm and its mass effect.

D. 49 year-old female with splenic pseudocyst (50*70 mm)

E. 48 year-old female with left paracolic cyst (80 mm), Right pelvic cyst (70 mm), retro bladder and antrorectal cyst (100 mm) and splenic cyst (100 mm).

 However, this significant relationship disappeared when the studied subjects were homogenized in terms of age. There was no significant correlation between serum vit D level and high blood pressure of black people in his study (20). Scragg et al. in 2007 emphasized the strong inverse relationship between blood pressure and the level of vit D in the elderly people according to the National Nutrition and Health Reviews (21). Several studies investigated the relationship between vit D and blood pressure (22, 23). Indirect effect of vit D on smooth muscles and vascular endothelial cells by activating the receptors of this vitamin and reduction of inflammatory process by vitamin D is a confirmed mechanism of action in reducing blood pressure (24, 25). Therefore, higher serum vit D is expected to be associated with lower blood pressure whereas in the present study, hypertensive patients unexpectedly show higher blood pressure and we found no association between vit D deficiency and hypertension. Nonetheless, some studies illustrated increased vascular resistance by vit D suggesting its stimulatory role in elevating blood pressure through changes in sensitivity of vascular cells (26, 27). He et al. found a positive relationship between vitamin D, PTH and blood pressure (27). In addition, in a review lower levels of vit D were observed in grade II hypertension and in healthy subjects with normal blood pressure, serum vit D correlated with blood pressure (28). Park et al, investigated the interaction of serum 25-hydroxy vit D levels and CYP1A1, CYP1B1 polymorphisms on blood pressure in an elderly population. The results showed that the genetic changes in CYP1A1, CYP1B1 and serum 25-OH D had synergy effect on blood pressure (29). Muray et al. found a positive relationship between vit D and blood pressure in certain genotypes of VDR (17). 

Similar to the present study, Margolis et al over a 7-year study period found increased systolic blood pressure by taking supplemental vit D and calcium in postmenopausal women (30). Nevertheless in another study Mateus et al. found a significant correlation between the serum level of PTH and hypertension but not with vitamin D (14). Discrepancies in relationship between serum vit D across various studies may be attributed to several factors including age, sex, ethnic characteristics, and prevalence of coexisted comorbidities paticularly HTN and vit D deficiency in the general population and the study groups. Additionally, several common chronic diseases such as vitamin D deficiency, diabetes, obesity, metabolic syndrome, and hypertension are prevalent in this geographic region (5, 8, 31, 32). These factors affect HTN and vitamin D status differently. In the present study, there was a direct relationship between serum vit D level and blood pressure considering the sensitivity of vit D receptor. In spite of that effect of VDR polymorphisms on blood pressure has not fully been confirmed in Iran yet (26). This study has limitations regarding inadequate sample size, lack of data about coexisting comorbidities and the associated factors of vit D deficiency, obesity, metabolic syndrome and many clinical conditions which are associated with vit D deficiency or affects serum vit D levels (31, 32). Therefore, the findings of this study require further epidemiological prospective longitudinal studies.

## Notes

**Funding:** This research was funded by vice-chancellery for Research and Technology of Babol University of Medical Sciences (No. 9032524).

## Citation:

Akbari R, Adelani B, Ghadimi R. Serum vitamin D in hypertensive patients versus healthy controls is there an association? Caspian J Intern Med 2016; 7(3): 168-172.
